# Characterization of the complete mitogenome of *Gammarus lacustris* (G.O. Sars, 1863) (Amphipoda: Gammaridae) and its phylogenetic position within Amphipoda

**DOI:** 10.1080/23802359.2021.1958083

**Published:** 2021-07-27

**Authors:** Jiasheng Li, Jianshe Zhou, Shiyi Chen, Haodi Shen, Ying Peng, Kun Zhang, Wenhua Huang, Xudong Liang, Bingjian Liu, Chi Zhang

**Affiliations:** a National Engineering Research Center for Marine Aquaculture, Zhejiang Ocean University, Zhoushan, China; b Institute of Fisheries Science, Tibet Academy of Agricultural and Animal Husbandry Sciences, Lhasa, P. R. China

**Keywords:** *Gammarus lacustris*, mitochondrial genome, phylogenetic relationships

## Abstract

*Gammarus lacustris* is native to the Qinghai–Tibet Plateau (QTP), widely distributed in alpine lakes. The complete mitochondrial DNA sequence of *G. lacustris* was 15,349 base pairs in length and comprised 13 protein-coding genes, 22 transfer RNA genes, 2 ribosomal RNA genes, and 1 control region. The BI tree showed that *G. lacustris* was most closely related to *Gammarus duebeni*, and indicated that *Gammarus*, *Gmelinoides*, *Brachyuropus*, *Pallaseopsis*, and *Eulimnogammarus* evolved from a common ancestor. The mitogenome of *G. lacustris* provides new molecular data for further taxonomic and phylogenetic studies of Amphipoda.

*Gammarus lacustris* (Amphipoda: Gammaridae) is a freshwater species, which is native to the Qinghai-Tibet Plateau (QTP) and widely distributed in alpine lakes (Hou and Li 2018). *Gammarus lacustris* has been found in Tibetan areas at altitudes of more than 5000 m, highly adapted to the extreme environment, such as strong UV radiation and hypoxia (Clewing et al. 2016). *Gammarus lacustris* is the food source for fishes and birds and maintains parasite diversity, playing an important role in the lacustrine food web (Shaw et al. 2020). The complete mitochondrial genome of *G. lacustris* provides an important resource for phylogenetic relationships and studying molecular evolution.

The *G. lacustris* sample was collected from Cuomujiri Lake (N 30.77°, E 90.79°), Tibet, Chian, and was stored in National Engineering Research Center for Marine Aquaculture, Zhejiang Ocean University (Jian, Chen, and 1522490198@qq.com) under the voucher number GL20200610. Total genomic DNA was extracted from muscle using the DNeasy tissue kit (Qiagen). The mitochondrial sequences, amplified by PCR with 17 pairs of primer (Table S1), were obtained through Sanger dideoxy sequencing and assembled by CodonCode Aligner 5.1.5. The assembled mitochondrial genome was annotated using the online tool MITOS (Bernt et al. 2013). The annotated sequence was deposited in GenBank with the accession number MZ029704.

Similar to the mitogenomes of *Gammarus*, the complete mitochondrial genome of *G. lacustris* was 15,349 bp in length, including 13 protein-coding genes (PCGs), 22 tRNA genes, 2 rRNA genes (16S and 12S), and a control region (CR) (Cormier et al. 2018). The overall contents of A, T, C, and G were 31.83%, 32.55%, 22.58%, and 13.04%, respectively, with a high AT bias (64.38%). Both AT-skew and GC-skew of the mitogenome were negative, −0.0111 and −0.2643, respectively. The proportion of coding sequences with a total length of 11,066 bp was 72.10%, and 3679 amino acids were encoded. Except for four PSGs (ND1, ND4, ND4L, and ND5) and ten tRNA genes (Tyr, Gln, Cys, Phe, His, Pro, Leu1, Leu2, Val, and Ser), other mitochondrial genes were encoded on the H-strand. The lengths of 16S ribosomal RNA and 12S ribosomal RNA were 980 bp and 750 bp, respectively, which were both located in the positions between tRNA-Leu and CR, being separated by tRNA-Val. The length of CR was 984 bp, ranging from 14,366 bp to 15,349 bp.

The phylogenetic relationships of *G. lacustris* within Amphipoda were reconstructed based on the 13 PSGs using the Bayesian inference (BI) phylogenetic tree. The BI tree was constructed by the software MrBayes 3.2.6 (Ronquist and Huelsenbeck 2003), with GTR + F + I + G4 as the best-fit evolutionary model determined by ModelFinder (Kalyaanamoorthy et al. 2017). The BI tree indicated that *G. lacustris* shared a close relationship with *Gammarus duebeni* (Figure 1). In addition, *Gammarus* species together with *Gmelinoides fasciatus*, *Brachyuropus grewingkii*, *Pallaseopsis kessleri*, and *Eulimnogammarus vittatus* formed a clade, representing these species evolved from a common ancestor (Figure 1). The complete mitogenome of *G. lacustris* presents here provides valuable resources for investigating the phylogenetic relationships within Amphipoda.

## Data Availability

The genome sequence data that support the findings of this study are openly available in GenBank of NCBI at (https://www.ncbi.nlm.nih.gov/nuccore/MZ029704) under the accession number: MZ029704. Phylogenetic analysis based on the sequences of the 13 PCGs in the mitogenome. BI posterior probabilities with 10,000 generations were shown next to nodes. The number after the species name was the GenBank accession number. The genome sequence in this study is labeled with a star.
